# Supplementary figures and images for: Mistreatment of newborns after childbirth in health facilities in Nepal: Results from a prospective cohort observational study
Source: PLoS One. 2021 Feb 17;16(2):e0246352. doi: 10.1371/journal.pone.0246352 (PMC7888656; doi:10.1371/journal.pone.0246352)

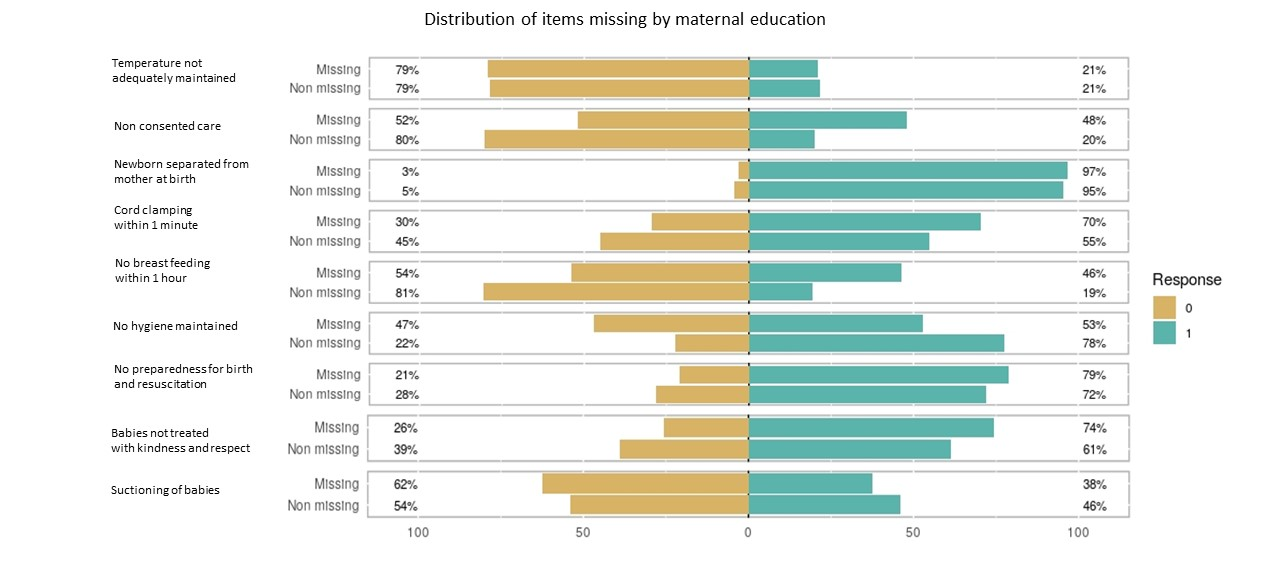

Supplement: S1 Fig — (TIF) [file pone.0246352.s003.tif]

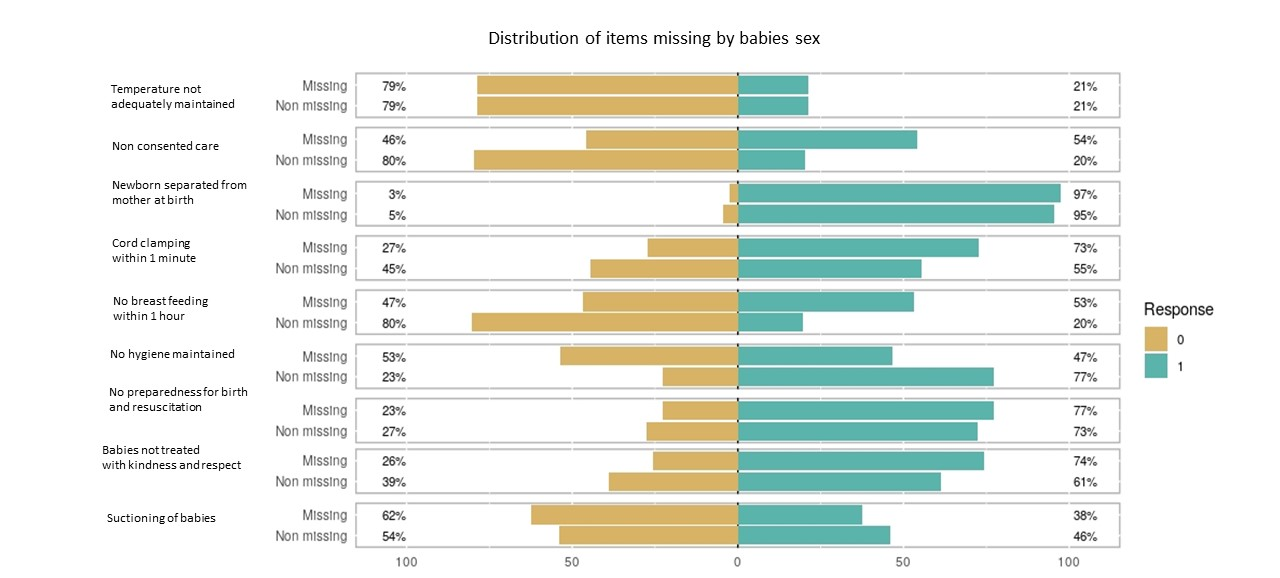

Supplement: S2 Fig — (TIF) [file pone.0246352.s004.tif]

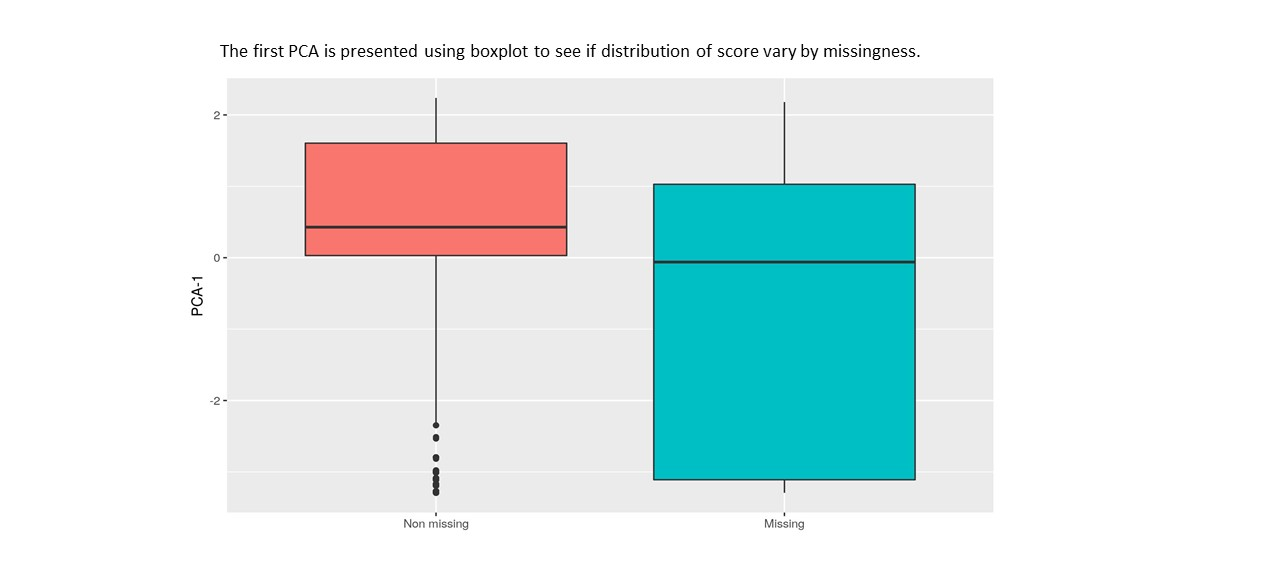

Supplement: S3 Fig — (TIF) [file pone.0246352.s005.tif]

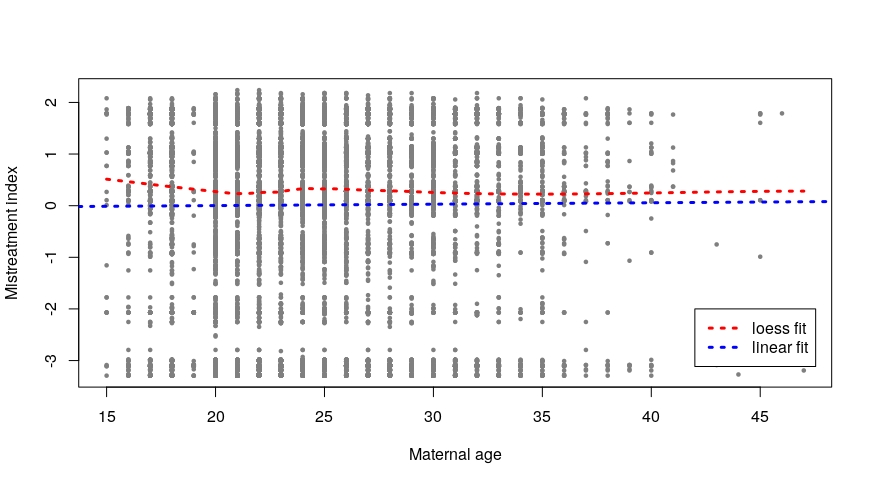

Supplement: S4 Fig — (TIF) [file pone.0246352.s006.tif]

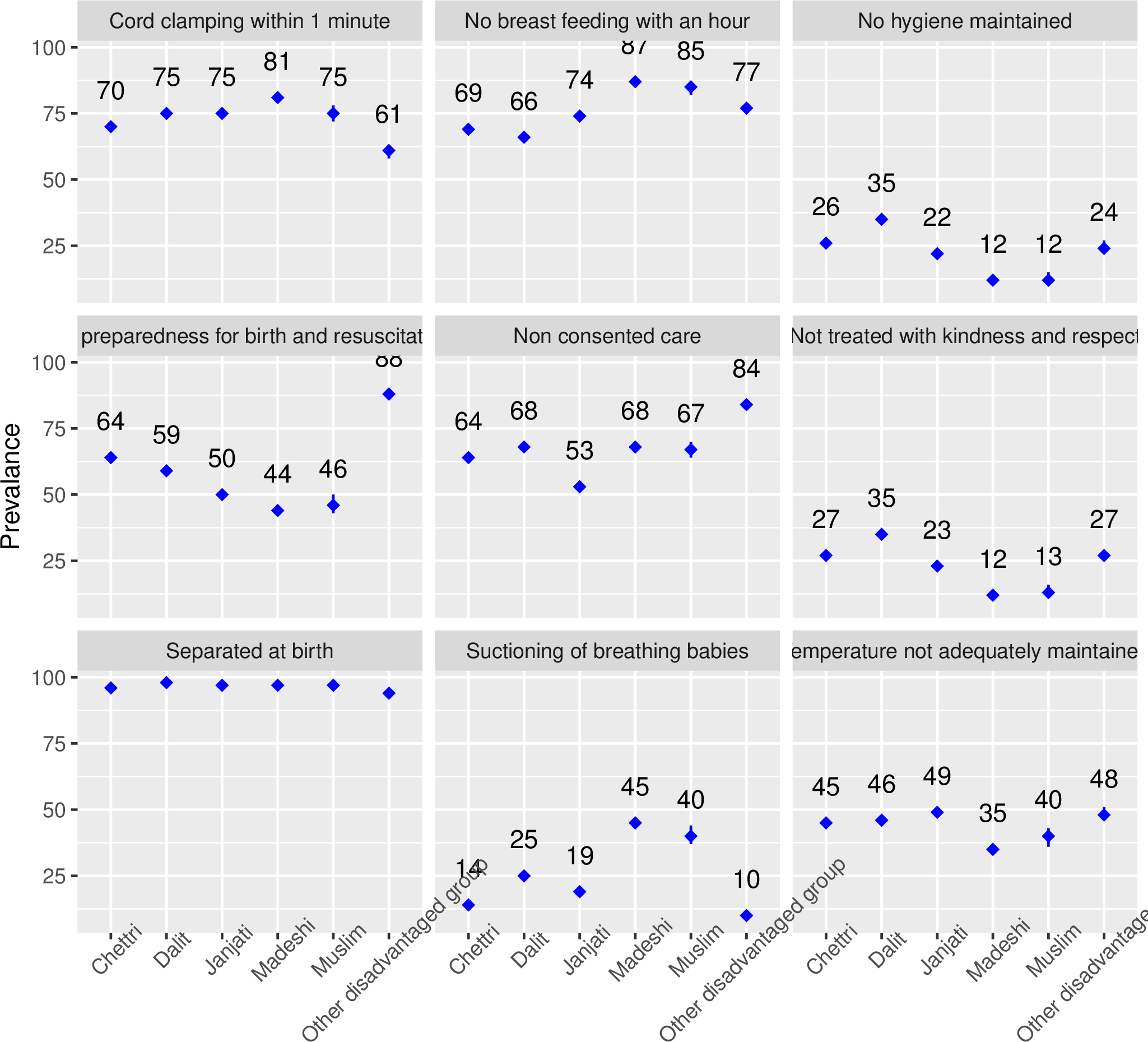

Supplement: S5 Fig — (TIF) [file pone.0246352.s007.tif]

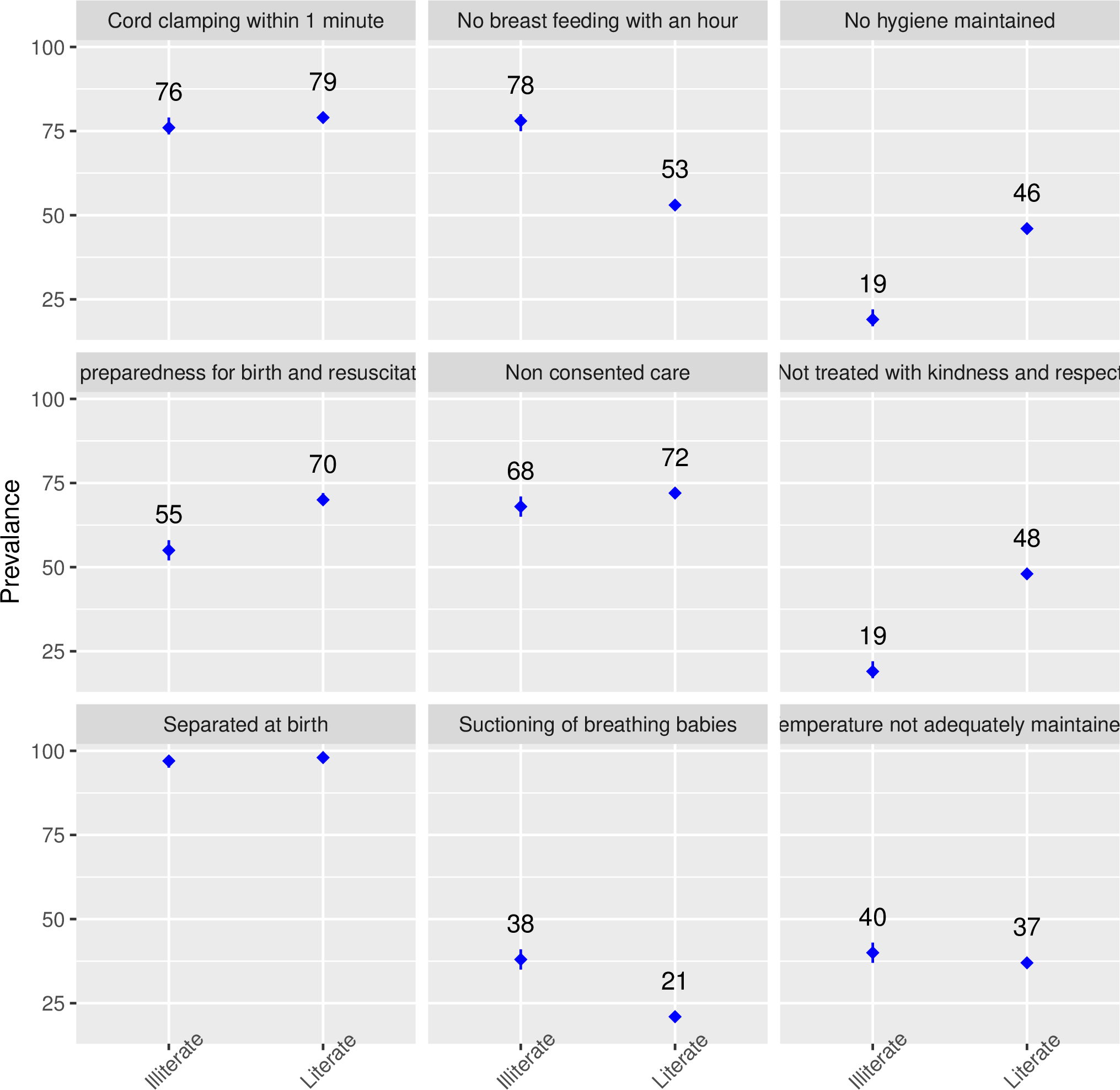

Supplement: S6 Fig — (TIF) [file pone.0246352.s008.tif]

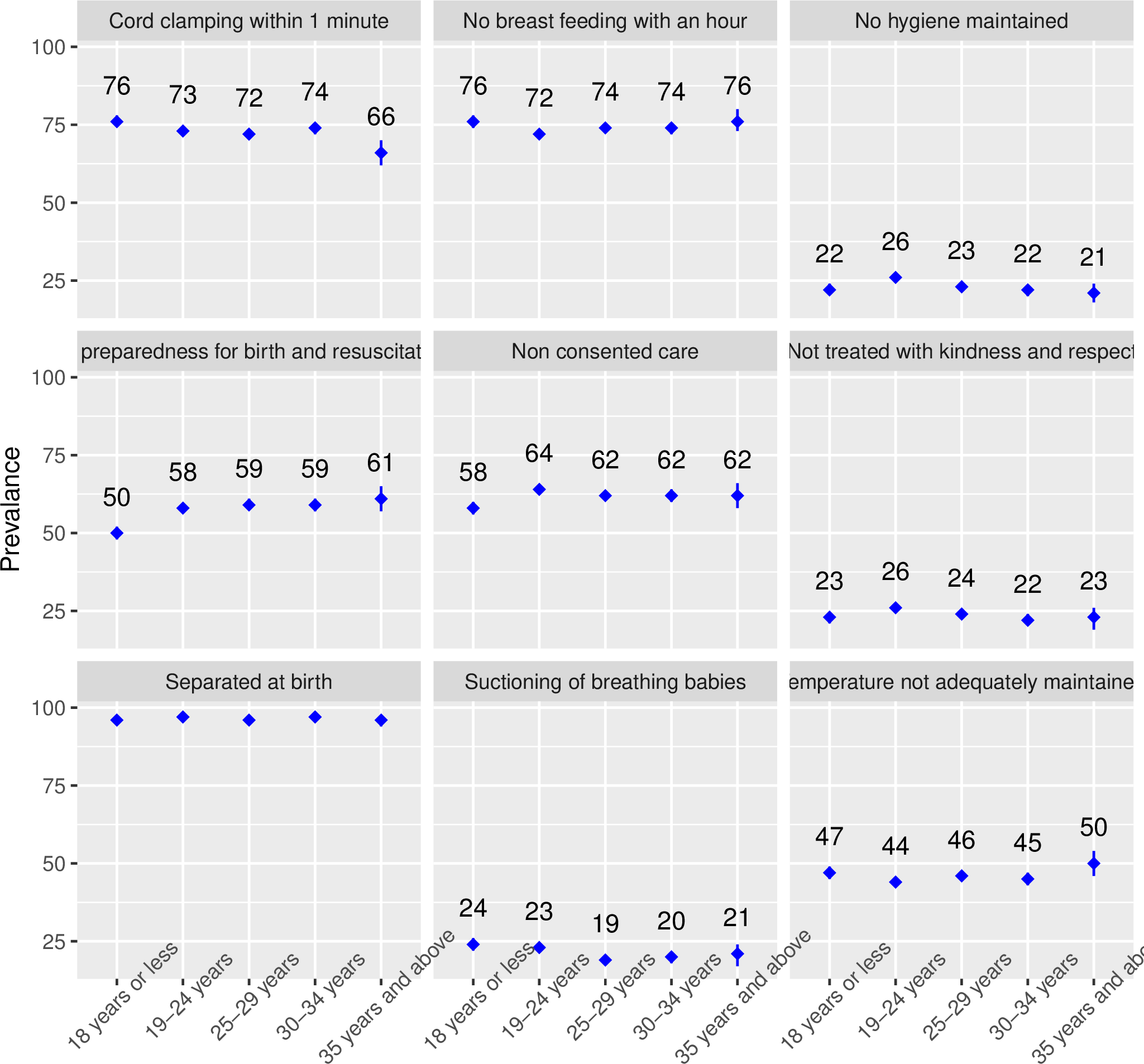

Supplement: S7 Fig — (TIF) [file pone.0246352.s009.tif]

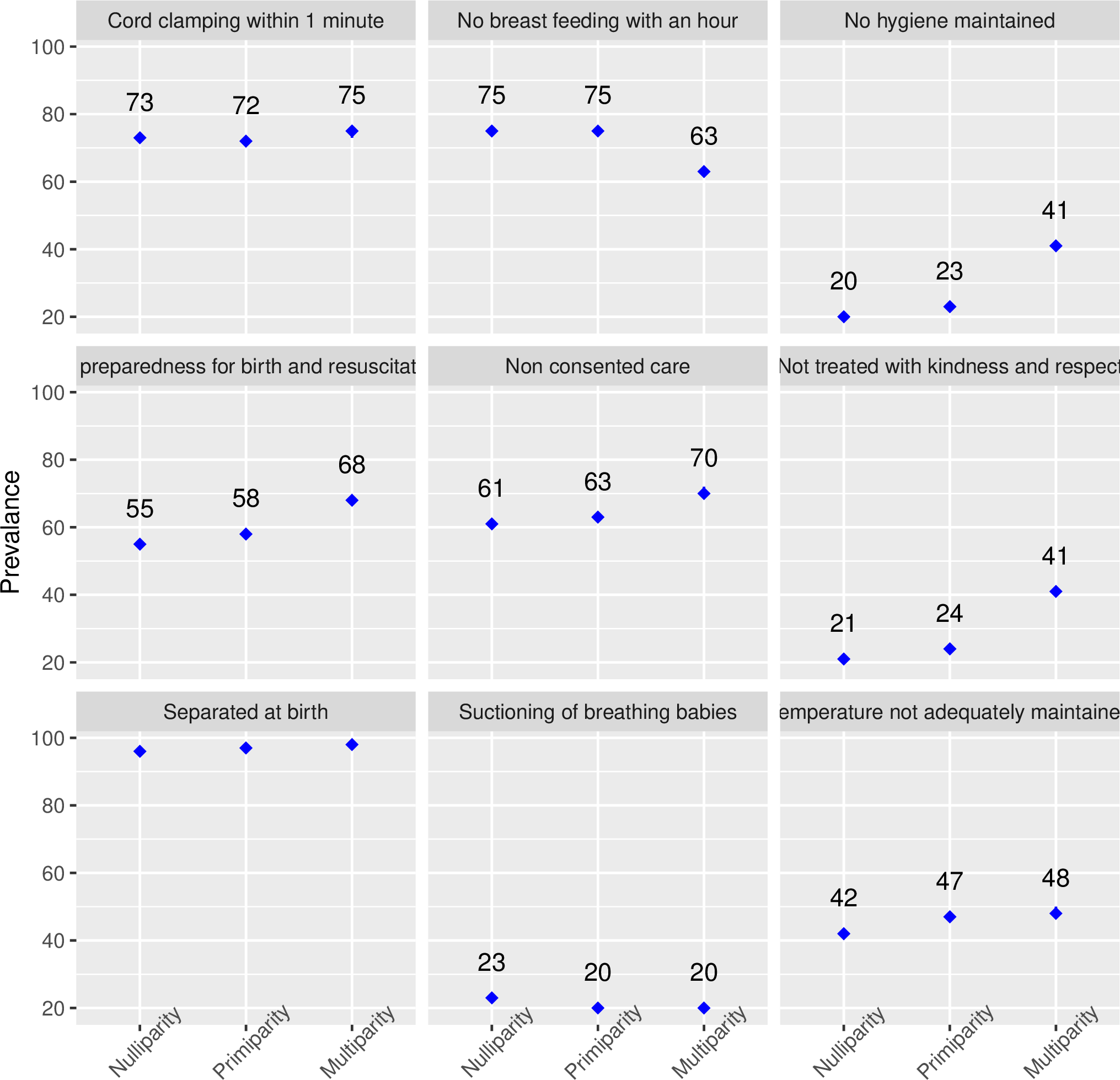

Supplement: S8 Fig — (TIF) [file pone.0246352.s010.tif]

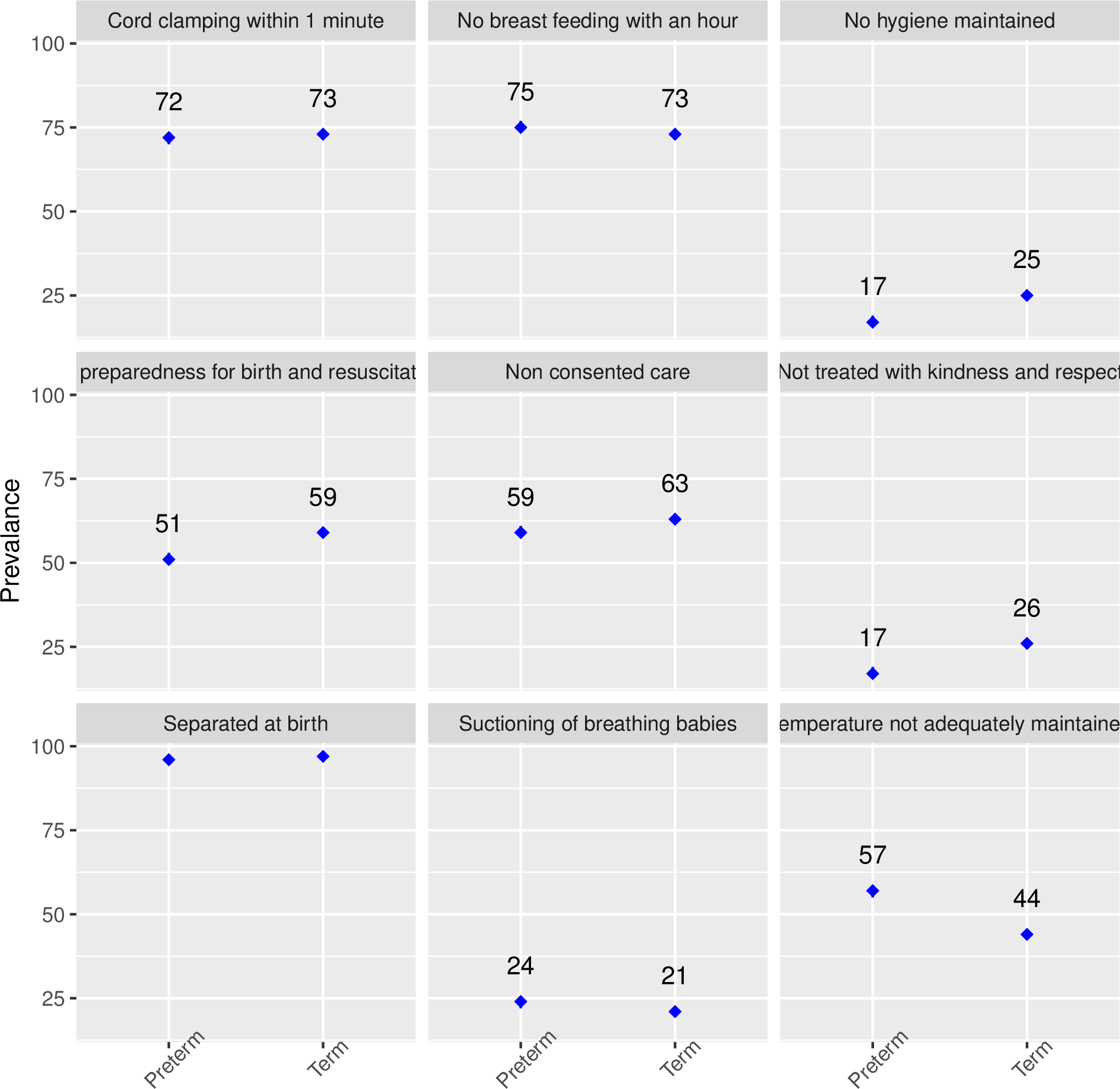

Supplement: S9 Fig — (TIF) [file pone.0246352.s011.tif]

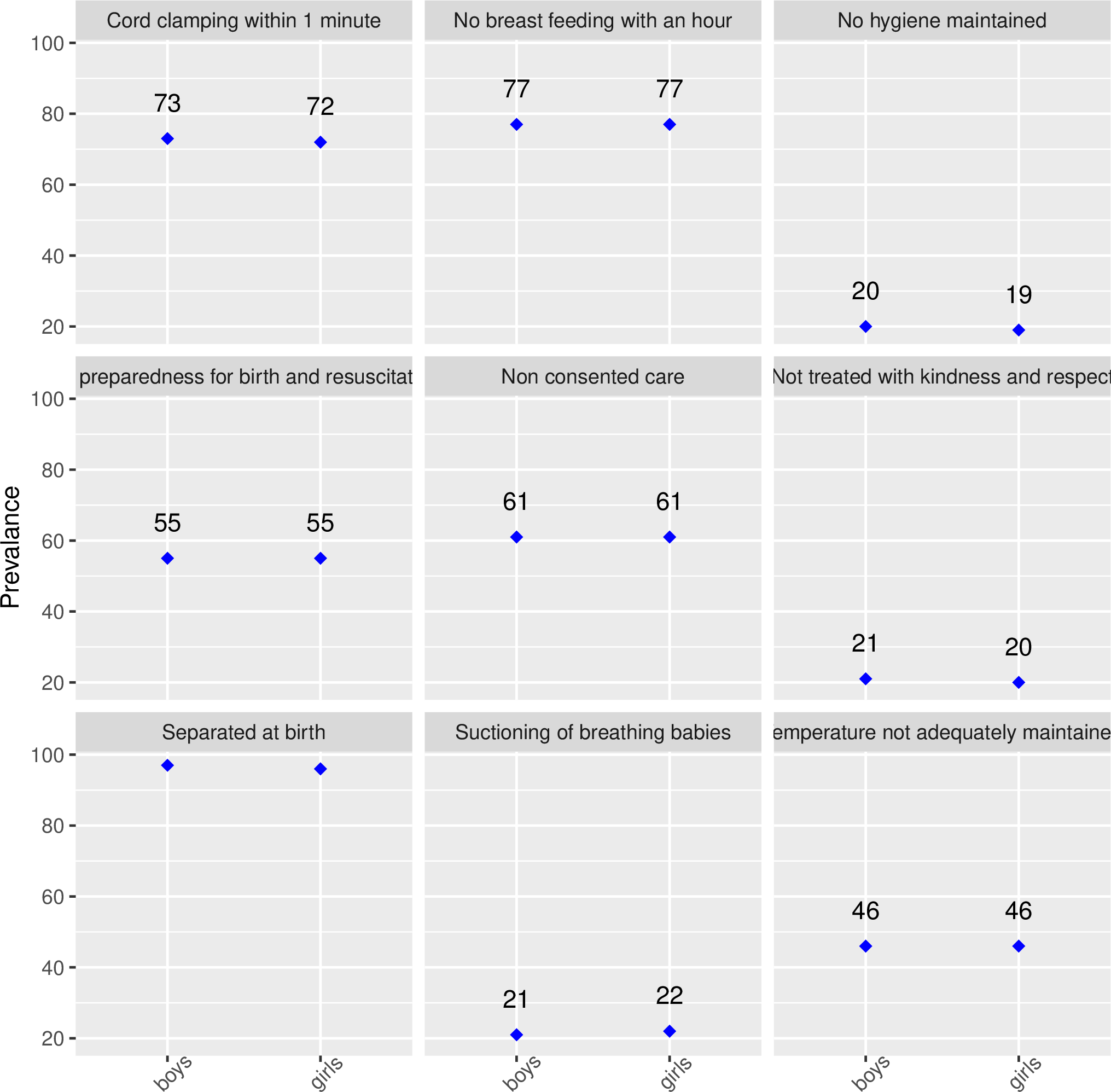

Supplement: S10 Fig — (TIF) [file pone.0246352.s012.tif]
