# Supplementary material for: Mistreatment of newborns after childbirth in health facilities in Nepal: Results from a prospective cohort observational study
Source: PLoS One. 2021 Feb 17;16(2):e0246352. doi: 10.1371/journal.pone.0246352 (PMC7888656; doi:10.1371/journal.pone.0246352)
Supplement: S1 Table — (DOCX) [file pone.0246352.s013.docx]

**S1 table.** **Definition of mistreatment among newborns during childbirth**

(Based on the WHO’s 2016 Standard for maternal and newborn care in health facilities)

| Standard | Quality Statement | Measure (number) | **Mistreatment** |
| --- | --- | --- | --- |
| Standard 1. Every woman and newborn receives routine, evidence-based care and management of complications during labour, childbirth and the early postnatal period, according to WHO guidelines | Quality statement 1.1b. Newborns receive routine care immediately after birth | Input measure-The health facility has local arrangements and a mechanism to maintain a documented room temperature in the labour and childbirth areas at or above 25 °C and free of draughts. (77.9) | Newborns kept in a room at a temperature less than 25 C |
|  |  | Output 1. Measure- The Proportion of all newborns who were breastfed within 1 hour of birth (*(86.42)* | Newborns who were not put to breast within 1 hour of birth |
|  |  | Output 2. Measure-The proportion of all newborns who were kept in skin-to-skin contact (with body and head covered) with their mothers for at least 1 hour after birth. *(83.34)* | Unnecessary separation of mother and newborn, such as lack of skin to skin contact |
|  |  | Output 3. Measure-The proportion of all newborns whose umbilical cord was clamped 1-3 minutes after birth *(76.56)* | Newborns who did not have their umbilical cords kept intact for at least 1 minute after birth |
|  | Quality statement 1.5-Newborns who are not breathing spontaneously receive appropriate stimulation and resuscitation with a bag-and-mask within 1 minute of birth, according to WHO guidelines. | Input measure 1. The health facility has a suction device, at least two sizes of neonatal mask and a self-inflating bag in the childbirth and neonatal areas of the maternity unit*. (93.83) | Inadequate preparation for birth and potential resuscitation |
|  |  | Output 1. Measure-The proportion of all newborns who were not breathing spontaneously after additional stimulation at the health facility who were resuscitated with a bag-and-mask. (81.10) | Newborns who were handled roughly and not treated with kindness and respect. Defined as “- spontaneously breathing newborns rubbed roughly and vigorously for extra-stimulation more times than recommended”. |
|  | Quality statement 1.8-All women and newborns receive care that includes standard precautions for preventing hospital-acquired infections | Output 1. measure-The percentage of health care staff in the health facility who clean their hands clean their hands correctly as per the WHO "5 moments for hand hygiene" audit tool *(87.78)* | 8. Newborns delivered in a non-hygienic conditions. |
|  | Quality statement 1.9-No woman or newborn is subjected to unnecessary or harmful practices during labour, childbirth and the early postnatal period | Output 6 indicator -The proportion of all babies born through clear amniotic fluid in the health facility who received routine suctioning (77.35) | Suctioning of newborns who did not require the intervention, such as those who were spontaneously breathing |
| **Standard 5 : Women and newborns receive care with respect and preservation of their dignity** | Quality statement 5.3: All women can make informed choices about the services they receive, and the reasons for interventions or outcomes are clearly explained. | Output measure 2- The proportion of all women who gave birth in the health facility who felt adequately informed by health care staff regarding decisions taken about their care. (80.82) | 5. Newborns who received medical care non-consented by a parent or guardian |
