# Supplementary material for: Mistreatment of newborns after childbirth in health facilities in Nepal: Results from a prospective cohort observational study
Source: PLoS One. 2021 Feb 17;16(2):e0246352. doi: 10.1371/journal.pone.0246352 (PMC7888656; doi:10.1371/journal.pone.0246352)
Supplement: S2 Table — (DOCX) [file pone.0246352.s014.docx]

S2 Table. Multi-level regression analysis on mistreatment among newborns (hospital and observer as random variable)

|  | Adjusting hospital level heterogeneity | | Adjusting observer level heterogeneity | |
| --- | --- | --- | --- | --- |
|  | Adjusted Beta | P-value | Adjusted Beta | P-value |
| (Intercept) | 0.0510 | 0.889 | 0.05752 | 0.95355 |
| Maternal Age | -0.011 | 0.009 | -0.01098 | 0.012389 |
| Maternal age (knot at 21 year) | 0.0166 | 0.0135 | 0.015205 | 0.025446 |
| Maternal age (knot at 24 year) | -0.00754 | 0.08735 | -0.00633 | 0.154731 |
| Ethnicity |  |  |  |  |
| Relatively advantaged ethnic group (Brahmin) | Reference |  | Reference |  |
| Dalit | 0.001486 | 0.889 | -0.00149 | 0.89043 |
| Janajati | -0.04227 | 0.001 | -0.0484 | 0.000445 |
| Madhesi | -0.02398 | 0.289 | -0.02665 | 0.24405 |
| Muslim | -0.00717 | 0.494 | -0.00886 | 0.403238 |
| Chhetri | 0.077727 | <0.001 | 0.075328 | 4.51E-05 |
| Parity |  |  |  |  |
| 0 previous birth | Reference |  | Reference |  |
| 1 previous birth | 0.00086 | 0.917 | 0.00616 | 0.442538 |
| 2-5 previous births | 0.060556 | <0.001 | 0.009662 | 0.361961 |
| **Sex** |  |  |  |  |
| Male | Reference |  | Reference |  |
| Female | 0.01099 | 0.095 | 0.011609 | 0.08144 |
| **Preterm** |  |  |  |  |
| Term | Reference |  | Reference |  |
| Preterm | 0.010013 | 0.360 | 0.008818 | 0.422852 |
| **Illiteracy** |  |  |  |  |
| Literate | Reference |  | Reference |  |
| Illiterate | -0.00561 | 0.621 | -0.00426 | 0.709926 |
