# Supplementary material for: Mistreatment of newborns after childbirth in health facilities in Nepal: Results from a prospective cohort observational study
Source: PLoS One. 2021 Feb 17;16(2):e0246352. doi: 10.1371/journal.pone.0246352 (PMC7888656; doi:10.1371/journal.pone.0246352)
Supplement: Attachment — Submitted filename: Response to editor and reviewers comment_21.05.2020.docx [file pone.0246352.s016.docx]

21 May 2020

**Response to editorial comment**

**Comment 1#** *We'd suggest (minorly) revising the title according to PLOS Medicine's style - the part after the colon, with the study design, would be better given as "prospective cohort" perhaps rather than simply prospective observational study?

Response- We have now added “prospective cohort” in the title.

**Comment 2#** *Minor rephrase of headings in abstract - Background, Methods and Findings, Conclusions.

Response-We have now revised it.

**Comment 3#***In the abstract, the reviewers (particularly reviewers #2 and #3) are asking for substantial more detail and clarity about some of the study methods and findings. We'd suggest attending to these points carefully, for example clarifying precise study dates, settings, who collected key outcomes data, and other crucial details.

Response- We have made revision and response to second and third reviewer’s comment on the methods and findings.

**Comment 4#***In the last sentence of the Abstract Methods and Findings section, please describe the main limitation(s) of the study's methodology.

Response- We have added the two major limitations in the abstract in page 3, lines 11-13 as “Two major limitations of the study were that the observation was only done for newborns born by vaginal deliveries and not by caesarean section and there might have been observers’ reporting bias.”

**Comment 5#***In the discussion section of the paper, key limitations are mentioned, which is good. However, the authors mention possible observer reporting bias but it's not made clear what direction this bias might tend towards and why, and it would be good to discuss this more. For example, outcomes data were collected by research nurses (is this correct?) - for outcomes such as "newborn treated roughly" or "received medical care non-consented", one might think that (even in non busy periods) it would be difficult for a healthcare professional to report that these outcomes were the case. (And that collecting such outcomes by asking caregivers to self-report anonymously might be more reliable). These factors could be discussed in more detail than is currently the case.

Response- We agree that some of the mistreatment observations done by research nurses such as “newborn treated roughly” and “received non-consented medical” would have been much accurately collected if collected anonymously through caregiver self-report following discharge in non-bias environment, however, this was not possible in this setting at the time of the study. If bias existed, we believe it would be as underreporting: nurses may have been less likely to report mistreatment events, and observations conducted during the busy clinical hours might also have been less likely to be recorded. We have added in the limitations in page 22, lines 11-17 as “Second, there might have been observer reporting bias, especially when the caseload for births was high, possibly resulting in fewer observations of mistreatments. Periodic quality assessment was done to reduce the bias. Third, there could have been some bias of reporting from the research nurses of the mistreatment observations, especially for indicators, such as “newborn treated roughly” and “received non-consented medical.” It would be preferable to also collect data anonymously through caregiver self-report following discharge in a non-bias environment.”

**Comment 6#***Did your study have a prospective protocol or analysis plan? Please state this (either way) early in the Methods section.

a) If a prospective analysis plan (from your funding proposal, IRB or other ethics committee submission, study protocol, or other planning document written before analyzing the data) was used in designing the study, please include the relevant prospectively written document with your revised manuscript as a Supporting Information file to be published alongside your study, and cite it in the Methods section. A legend for this file should be included at the end of your manuscript.

b) If no such document exists, please make sure that the Methods section transparently describes when analyses were planned, and when/why any data-driven changes to analyses took place.

c) In either case, changes in the analysis-- including those made in response to peer review comments-- should be identified as such in the Methods section of the paper, with rationale.

Response- This data collection on women and newborn care observation using the “WHO’s standard for maternal and newborn care in health facility” was part of a prospective study protocol. However, the analysis of mistreatment of newborn during childbirth was not part of the original study, which aimed to evaluate the care practice before and after quality improvement interventions. We have mentioned this in the method section in page 9, lines 9-14 as “The analysis of mistreatment of newborns during childbirth was not part of the original study, which aimed to evaluate the care practice before and after implementation of quality improvement interventions. These observations also captured elements of mistreatment of newborns. The data collection on maternal and newborn care observation using the “WHO’s standard for maternal and newborn care in health facility” [16] was part of the prospective study protocol.”

**Comment 7#***The authors already note that the paper is reported according to the STROBE guideline, however it would be ideal to also include the completed STROBE checklist as Supporting Information. 1 Please add the following statement, or similar, to the Methods: "This study is reported as per the Strengthening the Reporting of Observational Studies in Epidemiology (STROBE) guideline (Checklist)." The STROBE guideline can be found here: [http://www.equator-network.org/reporting-guidelines/strobe/.](http://www.equator-network.org/reporting-guidelines/strobe/) When completing the checklist, please use section and paragraph numbers, rather than page numbers.

Response- We have now provided the completed STROBE checklist as reported in the paper.

**Response to first reviewer’s comments**

**Comment 8#*** Age should not be categorized. Categorizing continuous independent variables is almost always a mistake. In *Regression Modeling Strategies* Frank Harrell lists 11 problems with this and summarizes "Nothing could be more disastrous".  Instead, leave age continuous and use a spline to investigate nonlinearities.

Response- We thank the reviewer for this comment. We agree that using maternal age as continuous variable is better than categorizing into age interval. Our statistician and co-author MM had examined maternal age to be used as a continuous variable and found non-linear association with mistreatment and different associations was seen at 21 and 24 year. To reduce the ambiguity of non-linearity, we kept age category interval. Further, from a public health intervention perspective, age interval (such as adolescent or elderly) interventions are feasible to implement. The texts in methodology in page 14, lines 22-24, page 15, lines 1-2 as “A non-linear association between maternal-age and the mistreatment index has been observed using Loess-smoothing on a scatter plot (supplementary figure 4) and two knots association was seen at 21 and 24 years using the broken-stick regression model (supplementary table 2). To avoid this ambiguity, linear regression was done for age category to assess the association with mistreatment index.”

**Comment 9#*** On p. 11, what are the 95% CI? From what model? Or are these quantiles of the actual variable?

Response-The 95% CI in table 1, is the quantiles of the actual variable We have now added in the description in page 13, line 9 as “* for the quantiles of the actual variable”

**Comment 10#*** Instead of (or maybe in addition to) hospital being a random variable, I think the person doing the evaluating should be a random variable.

Response- Thank you for this suggestion. We have now updated the model considering delivery observers (24 research nurses) as a random variable in. We did not consider both the delivery observer and hospital as random due to the model convergence issue. This is now mentioned in page 15, lines 7-10 as “This study was conducted in 4 hospitals and 24 trained data collectors have observed all the deliveries. Therefore, there is a possibility that the observations between the observers and between the hospitals are heterogeneous and within observers and within hospitals are correlated.” And lines 13-19 “First, we considered the hospital as random variable and employed a mixed model. Secondly, we considered observers as random variable. We also had a third model, where we considered observers nested within the hospital as random variable. In the third model, there were three different levels (birth, observer and hospital), so the model became complicated and we were unable to get the convergence and The outcome from the first and second model is presented in table 3 and the outcome of the first model has been reported in the main text.”

**Comment 11#*** The authors describe a principal component analysis. But, really, what they are after is a factor analysis. Especially with only one factor, it is unlikely to matter much, but the terminology is better as factor analysis. The goal of factor analysis is to estimate a latent variable, and that is what the authors were trying to do.

Response- Factor analysis and PCA are both methods to reduce data in order to get a single variable to represent the phenomena at hand. The methods have the same goal but reaches this goal differently, basically by having opposite directions in the analysis. The methods are also applicable when the theoretical assumptions are different. FA departs in the latent variable and assumes that this underlying (latent) variable is “causing” the responses in the variables observed, as in the case of anxiety for example. PCA goes the other direction, aiming to create a representative score through aggregating variables in a weighted manner. The most commonly used example is the wealth index, where different assets are aggregated into a measurement of “wealth”. In this study we have used a PCA to create a aggregated score for respectful newborn care and not a factor analysis, since we our theoretical assumption is that many different routines and work place culture outcomes result in disrespectful or respectful care and not the other way around. (ref <https://www.theanalysisfactor.com/the-fundamental-difference-between-principal-component-analysis-and-factor-analysis/>). Text in methodology is updated in page 13, lines 13-16 and page 14, lines 1-5 as “The usual practice is to weight the variable according to the first principal component i.e the component which has the highest variance, and thus the highest discriminatory power. This single index similar to a single factor in factor analysis setting. The continuous score is more flexible to analyse and to model. We consider the first principal component as the proxy for the mistreatment index as it explains more than thirty percent of the total variation in the mistreatment measure.”

**Comment 12#*** In Table 2, how were the variables parmaterized? Why is there an intercept for each variable?  I don't think I've ever seen that or seen a parameterization that would yield that. Effect coding? Helmert coding? Or what?

Response- Thank you very much for raising this issue. For the unadjusted model, we have considered the model $E[Y|x] = \alpha + \beta x$, where $x$ is the covaiate. In our case, we have six covariates and we have estimated this model for each covariate separately(unadjusted case). Therefore, for each model, we have an estimate of $\alpha$. On the other hand, for the multiple regression(adjusted) we have included all the covariates at a time and we have only one estimate of $\alpha$.

**Response to second reviewer’s comments**

**Comment 13#*** It would be helpful if the authors could clarify how a few of the key variables were operationalized and consistently assessed across locations. In particular, 'roughly handled' seems to be a very subjective concept, and it would be helpful if the authors could explain how this was conceptualized and what observers were trained to look for. Similarly, babies 'born in non-hygienic conditions' - what does that mean?

Response- Thank you for raising this issue. We have clarified definitions in the text and added a supplementary table in page 11, lines 16-20 as “The indicators selected was based on the WHO’s 2016 “Standards for improving quality of maternal and newborn care in health facilities” quality of care statement and process of care[16]. Deviation in the process of care for newborns during childbirth as stated in the guideline was defined as mistreatment for newborns (supplementary table 1).”

We defined the newborns who were handled roughly and not treated with kindness and respect as in page 12, lines 3-5 “spontaneously breathing newborns rubbed roughly and vigorously for extra-stimulation more times than recommended”.

We defined newborns delivered in a non-hygienic conditions in lines 13-15 as “Defined as health care staff who did not clean their hands clean their hands correctly as per the WHO’s five moments for hand hygiene”

**Comment 14*#** The issue of consent is also extremely interesting - do women ever give blanket consent for their baby's care? Or are they expected to individually consent to each procedure? Some might argue that exigent circumstances warrant waiving the normal requirements for consent, so it might be helpful to describe the types of procedures that women are not asked about and the types of circumstances in which such things occurred.

Response-The women usually give a blanket consent for their baby’s routine care. However, if there are additional procedures such as resuscitation, additional content is taken.

**Comment 15#*** Were there observers onsite 24 hours a day, 7 days a week? Or were there gaps in observations? How many nurse observers were assigned to each facility? Was each observer assigned to a unique delivery and follow women from arrival to post-partum, or were there hand-offs in observations? More detail on the process would be helpful, as well as the potential implications (both strengths and weaknesses) associated with whatever methods were chosen. This raises a separate question of how consistency across facilities was maintained for such an enormous volume of observations. Were observers periodically brought together for refresher trainings?

Response- We have now added the description of the observation process and the quality control mechanism to ensure consistency of observation made in the different hospitals. In page 11, lines 2-9 as “A standard operating protocol was developed for data collection and management. A week training was provided to the research nurses on the observation and supervised field observation was done by expert clinicians. Every two weeks, each research nurse team were provided feedback on the observation made based on the filled observation checklist. A quarterly data quality assurance assessment was conducted by the central level investigation team to assess adherence to the SOP. Feedback was provided to the data collection team on the adherence to the SOPs.”

**Comment 16#*** The authors point out that all covariates were found to be significantly associated with the mistreatment index in multivariate analysis, but a sample of 31,804 births leaves the possibility of lots of things being 'significant' by chance. The implications of such a large sample size should be discussed in the limitations section.

Response- We agree that a model with multiple potential co-variates, the probability that one or more will be associated by chance increases. However, large sample size reduce the possibility of having association of co-variate with mistreatment index by chance.

**Comment 17#*** This is a picky point, but the abstract says that babies of nulliparous women were less likely to be mistreated. But if a woman is nulliparous, she can't have a baby. By definition, once she delivers, she is primiparous. I recognize what the authors are trying to say by categorizing women into those with no previous births (who are nulliparous until they deliver, and then they are primiparous) compared to those with one previous birth or 2 or more. And it makes sense when described in the depth of the manuscript, but a quick read of the abstract gave me pause. I might just say something like, "babies born to women with one previous birth and women with 2 or more previous births were less likely to be mistreated than babies born to first-time mothers."

Response- Since, we agree that there might be a misinterpretation that occur when saying that babies of nulliparity were less likely to be mistreated. We had defined it as those who did not have previous birth as nulliparous. For the easiness purpose, we will have now revised in page 13, line 1-3 as “Parity was categorized as women who had no previous births, at least one previous birth, and two or more previous births.”

**Response to third reviewer’s comments**

**Comment 18#*** Abstract-Unclear how exactly data were collected. Were women asked? Observations done? The abstract is not stand-alone.

Response- We have added in abstract page 2, line 14 as “observed”.

**Comment 19#*** Abstract-'Women' in this respect is strange, since all along you had wanted to place emphasis on the newborn.

Response- We have now revised the abstract, page 3, lines 1-3 as “In multi-variate regression analysis, women age 35 years or more were more likely to be mistreated (β, 0.466; p value, 0.000) in reference to 18 years or less.”

**Comment 20*#** Abstract-The finding with regard to 'nulliparous' and 'primiparous' is very strange and I look forward to reading the rest of the paper in this respect. It hints on a problem in classification.

Response- We have revised it in page 3, lines 3-5, “Infants born to women with one previous birth (β, -0.176, p=0.000) and women with 2 or more previous births (β, -0.953; p value, 0.000) were less likely to be mistreated than infants born to first-time mothers.”

**Comment 21*#** The adjusted finding would fit much better with present literature.

Response- Thank you

**Comment 22*#** Formatting of affiliations (in full) requires some attention. Seems strange to place 'Bellville' after 'Cape Town', imagine it should be the other way round, and often Bellville is left out altogether but up to the authors I suppose

Response-We have now revised it.

**Comment 23*#** Author’s summary-Quite puzzled about what this variation in multiparous women actually entails. What is the meaning of this so-called core findings the authors identify? Not worked out in the next paragraph either

Response-We have revised the author summary on “what did the researcher do and find” consistent with core findings in page 5, lines 20-22 and page 6, lines 1-3 as “After adjusting for hospital heterogeneity, infants born to women aged 30-34 years (β, -0.041; p value, 0.01) and aged 35 years or more (β, -0.064; p value, 0.029) were less to be mistreated than infants born to women aged 18 years or less. Infants born to women from relatively disadvantaged (chhetri) ethnic groups (β, 0.077; p value, 0.000) were more likely to be mistreated than those infants to women from relatively advantaged (brahmin) ethnic group.”

**Comment 24*#** Author’s summary-age not mentioned under the former question. All of this seems rather randomly written up to me.

Response-we have now revised it and mentioned it in response to comment 23.

**Comment 25*#** Main text- page 6, lines 2-3 “In the last 20 years, there has been an extraordinary rise of institutional deliveries with over two third of births now taking place in health facilities” Generally would prefer 'births' over 'deliveries' throughout as more women-centered terminology

Response- Thank you for this recommendation. We have now ensured women-centered terminology such as ‘birth’ over deliveries throughout the manuscript.

**Comment 26*#** Main text- page 6, lines 14-16 “However, several studies in low- and middle-income settings in South Asia and Sub-Saharan Africa have shown widespread disrespect and abuse of mothers during childbirth and in the postnatal period” quite a stigmatizing statement. It is not only about these settings. Look for instance at Respectful and disrespectful care in the Czech Republic: an online survey Cecily Begley, Natalie Sedlicka & Deirdre Daly. Reproductive Health. volume 15, Article number: 198 (2018)

Response-We have now revised the sentence in page 7, line 15 as “…reported disrespectful care to…”

**Comment 27*#** Main text-page 7, lines 13-15, “Based on the White Ribbon Alliance’s Respectful Maternity Care (RMC) typology for abuse and disrespect, category for respectful care of newborns were proposed in 2017” categories of? Or do you mean criteria for?.

Response- We have revised it in page 8, lines 13-15 “Based on the typology for abuse and disrespect during childbirth proposed in 2011 by Browser and Hill [13], respectful care of newborns were proposed in 2019 by White Ribbon Alliance [15]…….”

**Comment 28*#** Main text- since the study was not set up to specifically detect mistreatment, we have to be careful in attributing excessive value to the outcomes. If you would design a more specific study, you may in fact find higher levels of mistreatment and gain more insight into associated factors.

Response-Thank you for raising this issue. We are aware that the study was not primarily designed to detect mistreatment, but we believe that the observations made still gives a valid picture of the situation, and allows us to draw conclusions from it. Further studies that take this concept to further depth are warranted. We have added this to the limitation section in page 22, lines 18-20 “….. as not assess some health system constraints, such as number of women and babies taken care by each nurses per day as the study was not set up to detect mistreatment.”

**Comment 29*#** Main text- page 10, lines 2-3, “For the larger study, an estimated 80,000 women-baby pairs were required to assess the change in intrapartum related mortality” I wonder what happened to the other 40 000 not included in your study, why were the pairs selected that were selected? Were they the ones who were admitted for birth? Unclear

Response – In the larger study of 80000 women-baby in 12 hospitals, data collection through record review and pre-discharge interview was done to assess the change in the mortality. We have added this in page 11, lines 12-14 as “For the larger study, an estimated 80,000 women-baby pairs were required to assess the change in intrapartum related mortality[23]. No sample size calculations was required for the nested observation component.”

To assess the change in the clinical care practice, observation was done in 4 public hospitals over the period of 18 months, which enrolled 30,000 women-infant. This has been pre-specified in the study protocol <https://www.ncbi.nlm.nih.gov/pmc/articles/PMC5640082/pdf/bmjgh-2017-000497.pdf>

**Comment 30*#** Main text- page 10, line 6, “We used the following indicators to assess mistreatment:” what were these indicators based on? How did you arrive at them?

Response-We have now added the basis of the mistreatment for newborns. During the design of the larger QoC study, we used WHO’s 2016 guideline on “Quality of Care for mothers and newborns in health facilities”. We have mentioned this in page 11, lines 16-20 as “The indicators selected was based on the WHO’s 2016 “Standards for improving quality of maternal and newborn care in health facilities” quality of care statement and process of care[16]. Deviation in the process of care for newborns during childbirth as stated in the guideline was defined as mistreatment for newborns (supplementary table 1).”

**Comment 31*#** Main text-page 10, line 16, “Health system constraints” I could easily think of other ones (not having enough nurses to look after the no of children, etc etc). I suppose these were the ony ones assessed in the larger QoC study? This is a limitation

Response-We agree that we did not assess all the health system constraints, especially the number of nurses to look after number of women and babies in an average daily. We have now mentioned this in the limitation section in page 22, lines 20-22 as “Fourth, we did not assess some health system constraints such as number of women and babies taken care by each nurses per day.”

**Comment 32*#** Main text-page 11, line 6. This is problematic. 'Nulliparous' women strictly cannot have newborns that can be assessed according to your definition. However, you may mean that the newborns assessed were in fact there first children. This would make them 'primiparous' according to your definition. This is a common confusion in many reporting systems. In general, reporters mean the same by 'nulliparity' and 'primiparity'. Therefore, I am weary of the validity of what will be reported

Response-As per our definition for in the study, nulliparity was women with no previous birth, primiparity was women with one previous birth and multi-parity was women with two or more previous birth. For the simplicity of reporting and agreeing to the reviewer’s comment, we have renamed nulliparity as “women with no previous birth”, primi-parity as “women with one previous birth” and multiparity as “women with two or more previous birth”. We have mentioned in page 13 lines 1-2 “Parity was categorized as women who had no previous births, at least one previous birth, and two or more previous births.”

**Comment 33*#** Main text- page 12, lines 1-2. according to the authors I take it... I wonder whether -if you suction a breathing baby for instance- this is not always 'mistreatment'. Better would be of course to conduct some sort of Delphi to construct such an 'index'. In that way, the index would go beyond these arbitrary choices among the authors themselves.

Response- Thank you for raising this issue. We have now added the basis of the mistreatment for newborns in page 11, lines 17-20. During the design of the larger QoC study, we used WHO’s 2016 guideline on “Quality of Care for mothers and newborns in health facilities”. The guideline provides the quality statement together with input, process and outcome of the quality of care. Any deviance in the process of care for newborn during birth was considered as mistreatment in the study. We have now added it in page 11, lines 16-20 as “The indicators selected was based on the WHO’s 2016 “Standards for improving quality of maternal and newborn care in health facilities” quality of care statement and process of care[16]. Deviation in the process of care for newborns during childbirth as stated in the guideline was defined as mistreatment for newborns (supplementary table 1).”

**Comment 34*#** Main text- page 12, I can understand why authors chose to impute, although a large number of missings still comprises a study limitation

Response- There was large number of missing for maternal literacy and sex of newborn and the values missing were not distributed uniformly to exclude the missing value from analysis, so imputation was done. We have added missing value of women’s literacy and baby’s sex as limitation in page 22, line 22, page 23, lines 1-2 as “Finally, there were large numbers of missing values for maternal literacy and sex of newborn and the values missing were not distributed uniformly and randomly to exclude the missing value from analysis, so imputation was done.”

**Comment 35*#** Main text-page 13, line 6, “adjusting the hospital level heterogeneity” I can understand why authors chose to impute, although a large number of missings still comprises a study limitation in page 20, lines 21-22, page 21, line 1-2 as “Finally, there were large numbers of missing values for maternal literacy and sex of newborn and the values missing were not distributed uniformly and randomly to exclude the missing value from analysis, so imputation was done.”

Response- Yes, we have now added it as limitation.

**Comment 36*#** Main text- page 14, lines 10-12 “Maximum variation in mistreatment index was observed among the advantaged ethnic groups and multi-parous women (Figure 3).” the fact that nulli/primips are relatively similar and the multips are the ones who differ also make me weary of this outcome (were not some primips actually nulliparous women? Misclassification?). It would be much more logical if the difference were seen between any or no children prior to this newborn.

Response-We have revised the term nulli and primi para. The figure 3 is the variation of the mistreatment index across background characteristics-parity without adjusting with hospital level heterogeneity.

**Comment 37*#** Main text- page 14, lines 16-18 “Babies from the janjati ethnic group were more likely to be mistreated (β, 0.848; p value, 0.000) in reference to advantaged ethnic groups.” still unclear to me what the 'advantaged group' actually is. Did I overlook this?

Response-Thank you. We have now made clearer division of relatively disadvantaged and relatively advantaged ethnic group in the method section in page 12, lines 20-21 as “., relatively disadvantaged ethnic groups (chhetri, Janajati, Madeshi, Muslim, Dalit) and relatively advantaged ethnic group (Brahmin).”. We have added the terms in page 16, lines 21-23 as “ Infants born to women of relatively disadvantaged (janjati) ethnic group were more likely to be mistreated (β, 0.848; p value, 0.000) in reference to relatively advantaged (brahmin) ethnic groups.”

**Comment 38*#** Main text-page 15, table 2. “Regression analysis of mistreatment among newborn (using mistreatment index).” adjusted for all other factors I suppose? Not entirely clear to me how the model was built, but all these items are of course correlated so I acknowledge the need for multivariate modelling

Response- The regression analysis of mistreatment among newborn was done using potential covariates (age, literacy, ethnicity, parity, sex of baby and preterm status) and described in the methods section.

**Comment 39*#** Main text-page 15, line 3-4 “We adjusted the hospital heterogeneity through a random intercept model. The hospital specific intercepts (β) were -2.87, 1.54, 0.95 and 0.38 at hospitals 1, 2, 3 and 4 respectively.” So very different hospitals indeed.

Response- Yes, we have now added in the page 19, line 5 as “ and varies by health facilities, ethnicity, women’s age and sex of baby.” We have also mentioned it in the conclusion section in page 21 as “Further, studies will be required to investigate the variance of mistreatment by health facilities”

**Comment 40*#** Main text-page 16, lines 7-8 “Female newborns (β, 0.016; p value, 0.015) were more likely to be mistreated than male newborns (Table 3).” so the other differences were mostly explained by differences in hospital? This merits thorough reflection.

Response- We have a paragraph in page 21, lines 19-21 and have added the underlying reason for the discriminatory behaviour towards female newborns as “In Nepal, like in other South Asia, there is a strong male preference which has led to serious discriminatory behaviour towards female babies [35] even at the health institution settings as evident in this study.”

**Comment 41*#** Main text-page 17, lines 4-5 “The study reports on the prevalence of mistreatment among healthy facility-born newborns after birth.” These descriptive findings are indeed the most revealing. I think from the modelling the results become more fuzzy to me.

Response- Thank you. We hope that the edits to the text further explaining the variation in the mistreatment among newborn by health facility, age, ethnicity and sex of baby makes the results clearer.

**Comment 42*#** Main text-page 19, Limitation “Based on my comments above I discern a number of quite obvious additional limitations that must be discussed.”

We now added three limitations in page 22, lines 11-20. “Second, there might have been observer reporting bias, especially when the caseload for births was high, possibly resulting in fewer observations of mistreatments. Periodic quality assessment was done to reduce the bias. Third, there could have been some bias of reporting from the research nurses of the mistreatment observations, especially for indicators, such as “newborn treated roughly” and “received non-consented medical.” It would be preferable to also collect data anonymously through caregiver self-report following discharge in a non-bias environment. Fourth, we did not assess some health system constraints, such as number of women and babies taken care by each nurses per day.”
