# Supplementary material for: Mistreatment of newborns after childbirth in health facilities in Nepal: Results from a prospective cohort observational study
Source: PLoS One. 2021 Feb 17;16(2):e0246352. doi: 10.1371/journal.pone.0246352 (PMC7888656; doi:10.1371/journal.pone.0246352)
Supplement: Attachment — Submitted filename: Response to editors and reviewers_13.10.2020_clean version.docx [file pone.0246352.s017.docx]

13 October 2020

David Desseauve, MD, MPH, PhD
Academic Editor
PLOS ONE

**Subject- Revision of the manuscript and response to reviewer’s comment on manuscript PONE-D-20-21414 “Mistreatment among newborns after childbirth in health facilities in Nepal: results from a prospective cohort observational study”**

Dear Dr. Desseauve,

Thank you for reviewing the manuscript. We have now revised the manuscript with point by point response to editor’s and reviewers’ comment.

**Response to editor’s comment**

**Comment 1# -** Please first of all provide the accordance and the protocol of the ethics commission to clarify this crucial point.If applicable, we recommend that you deposit your laboratory protocols in protocols.io to enhance the reproducibility of your results. Protocols.io assigns your protocol its own identifier (DOI) so that it can be cited independently in the future. For instructions see: <http://journals.plos.org/plosone/s/submission-guidelines#loc-laboratory-protocols>


Response-We have attached the ethics approval provided by the ethics review committee of Nepal Health Research Council. We have also attached the study protocol published in BMJ protocol. Please note that this study is a nested study within a larger study that evaluated health worker’s performance on neonatal care. We use the observational data on health worker’s performance as per the protocol to assess various newborn care practices that constitute mistreatment. We have also deposited the data and data analysis protocol.

**Response to first reviewer’s comments**

**Comment 2#** General: the article is well and logically written, but it is too long and will gain in being more concise and straight, in particular: abstract (2 pages), author summary, introduction, discussion (4 pages)

**Response-** We have now shortened the abstract and body of the article.

**Comment 3#** Abstract: information on cord clamping, breast-feeding during the first hour and skin to skin contact would be important to report.

**Response-** We have added the information on cord clamping, breast-feeding during the first hour and skin to skin contact to the abstract.

**Comment 4#** Methods: more information about the four hospitals would be interesting (number of yearly deliveries, rates of C-section, number of midwifes, nurses and physicians)

**Response-** We have now added the more information in page 9, lines 17-22 and page 10, lines 1-2.

**Comment 5#** Statistics: complex and not easy to follow for a non-statistician. Anyway, it seems it could be interesting to add a composite dichotomic variable of “any” or “no” mistreatment to know which proportion of babies are concerned, even (or especially!) if very high.

**Response-** Thank you for noting this. We have simplified our explanation of the methods and have added a simple summary at the start of the results section. However, we have retained some explanations of the statistics as this is an academic journal and these details are required. We created a scoring index of mistreatment using the principal component analysis of the childbirth care related-indicators, and conducted linear regression based on the mistreatment index. Therefore, it will not be possible to dichotomize the mistreatment index.

**Comment 6#** Discussion, page 20, lines 20-21: “Newborns who were spontaneously breathing also received unnecessary and vigorous stimulation, which is not evidence-based”. In the methods, it is set that non spontaneously breathing was a criteria exclusion.

**Response-** Correct, newborns who were not spontaneously breathing were excluded as they require additional (e.g. not routine) care. We included only newborns who spontaneously breathed at birth. These newborns do not require any resuscitation i.e. stimulation. Therefore, providing unnecessary and vigorous stimulation to these spontaneously breathing newborns is not evidence-based.

**Comment 7#** Abstract, page 3, line2: “women age 35 or more were more likely to be mistreated”  “Infants born to women (?!)were more likely to be mistreated”

**Response-** Thank you. We have now revised it as “infants born to” women etc.

**Comment 8#** Abstract, results, pages 2-3 and author summary, pages 5-6: presentations of factors should be in the same way, either increasing or decreasing the risk of mistreatment, but mixing the two is somewhat confusing for the reader.

**Response-** We have now ensured that the presentations of factors in abstract and summary are presented in the same direction.

**Comment 9#** Method, page 14, lines 1-2: “The single index similar to a single factor analysis setting”: missing verb?

**Response-** We have now added the verb.

**Comment 10#** Method, page 15, line 15-19: “In the third model, there were three different levels (birth, observer and hospital), so the model became complicated and we were unable to get the convergence and The outcome from the first and second 18 model is presented in table 3 and the outcome of the first model has been reported in the 19 main text: missing punctuation?

**Response-** We have added a punctuation.

**Response to second reviewer’s comments**

**Comment 11#** The relationship of the here presented data to the initial study is not quite clear and leads to some major questions. Consent: It is not quite clear what was included in the consent obtained by the mothers. Some fundamental questions might arise and should be cleared before publishing this paper.

**Response-** We have clarified the questions on consent in the below queries.

**Comment 12#** One of the major results is the lack of consent for treatment issues in spontaneously breathing infants. It seems difficult to understand that mothers who consented for this study should be asked for additional consent for each treatment approach.

**Response**-Women consented to the larger study which was to be observed for research purposes by an independent observer, but this was different than the providers seeking consent for treatment or care (Of self or newborn).

**Comment 13#** Was stated in the consent form that the well-trained study nurse would not intervene in the treatment of the newborn infant, although this treatment might be misconducted and not performed according to the international standards?

**Response-** We recognize the challenge for clinicians in the role of non-participating observers. In this case, the trained study nurses were trained on clinical observation and recording and not on clinical skills to intervene in care during childbirth and immediately after birth. In condition of life-threatening emergency of women and newborn infant, the well-trained study nurses were trained to inform the clinicians.

**Comment 14#** Hospital selection? The data presented in the abstract demonstrate that the influence of hospitals might have an important impact on the results, as adjustment for hospital heterogeneity inverse the results from more to less mistreatment in women over 35 years.

**Response**- For the larger study, 23 hospitals were selected. It was a clustered study and the highest volume hospital in each study served as the site for observations. For this study, only those 4 hospitals where clinical observations were done were selected.

Since the intervention time in these hospitals differed from each other, there was variation in the quality of care. This is reflective in the results, as the results changed when hospitals were adjusted. The change in the result after adjusting the hospital heterogeneity indicates that the coverage of respectful care is not homogenous by hospital.

**Comment 15#** Abstract: Methods and Findings: In the second paragraph is stated: In multi-variate regression analysis, women age 35 years or more were more likely to be mistreated (β, 0.466; p value, 0.000) in reference to 18 years or less. In the next paragraph is stated: After adjusting for hospital heterogeneity, women aged 30-34 years (β, -0.041; p value, 0.01) and aged 35 years or more (β, -0.064; p value, 0.029) were less mistreated in reference to women aged 18 years or less. I could not find any explanation, why this study was performed only in four out of the twelve hospitals and how they have been selected.

**Response-** In abstract, to avoid the confusion, we presented only the results after adjusting with hospital heterogeneity. We removed the paragraph “In multi-variate regression analysis, women age 35 years or more were more likely to be mistreated (β, 0.466; p value, 0.000) in reference to 18 years or less.” We have explained in the response to comment 13 on why 4 out of 12 hospitals were selected.

**Comment 16#** Cesarean section: In Figure 1 the flow chart is given for the 39’652 women admitted to one of the four hospitals, leading to the 31’804 deliveries, which were observed. The abstract indicates as one major limitations that newborns delivered by caesarean section have not been included. The number of children born by CS should be given and substracted from the 31’804 deliveries in order to get the final denominator.

**Response-** The figure 1, 39,652 women are those women who were admitted in the labour room for vaginal birth for observation. This does not include women who were taken to operation theatre directly for caesarean section. Those women who were admitted to labour room and take to operation theatre for emergency caesarean section were excluded in the analysis. We have corrected the typo as “excluded, did not deliver in delivery room”

**Comment 17#** Evolution over time: The goal of the original study was to measure the impact of training regarding quality improvement in mother and newborn care. No information in the here presented study is given regarding the timely evolution over the 18-month study period. There is a potential study bias, which should unveiled.

Response- We agree the study is a nested study and the aim of the larger study was to impact quality improvement of a neonatal resuscitation intervention on health worker’s performance and birth outcome. the larger study was targeting non-spontaneous breathing infants and not expected to have an impact on routine care. However, we have noted the possible impact of change over time in the limitations. months has been mentioned in page 9, lines 5-7.

**Comment 18#** Methods: Design: April 17, 2017 – October 2018 = 19 months (exact data of study period should be stated).

Response- It is an 18 month study with time point 14 April 2017 and 17 October 2018. We have now added it.

**Comment #19:**

I do not comment on the questions and remarks of the other reviewers, which I fully agree with.

I would conclude that the study unveils important lack of newborn care in four Nepalese Hospitals which might point to similar problems in many other maternity wards worldwide.

There are systematic problems which should be taught differently:

o late cord clamping and breast feeding within the first hour seems not to be part of the teaching standards as it was not performed in > 73% of situations.

o Even more the fact that the well-being newborn is separated from the mother in > 95% of all vaginal deliveries.

o The question of informed consent needs to be approached differently.

o Temperature control in the delivery room is in 50% of the hospitals not given.

o Preparedness and hygiene is important to increase

Therefore, the information of this study is important. However, the abovementioned major questions should be answered before publication.

Response- Thank you. We have endevoured to answer the above questions from reviewers, as we also believe this study has important findings for the Nepalese health system and newborns.

**Comment #20** PLOS authors have the option to publish the peer review history of their article (what does this mean?). If published, this will include your full peer review and any attached files.

If you choose “no”, your identity will remain anonymous but your review may still be made public.

**Response**- We are fine with the publication of the peer review history for this manuscript.
