# Supplementary material for: Mistreatment of newborns after childbirth in health facilities in Nepal: Results from a prospective cohort observational study
Source: PLoS One. 2021 Feb 17;16(2):e0246352. doi: 10.1371/journal.pone.0246352 (PMC7888656; doi:10.1371/journal.pone.0246352)
Supplement: Attachment — Submitted filename: Response to reviewers comment 28.12.20.docx [file pone.0246352.s018.docx]

**28 December 2020**

**Response to first reviewer’s comment**

**Comment 1#.** more emphasis could be brought on the most frequent mistreatment, esp lack of skin to skin contact immediately after birth that concerns almost all of the infants and could be easy to improve; what was the reason/barrier for this delay?

**Response.** The lack of skin to skin contact immediately after birth might be due to health workers lack of knowledge on the importance of skin to skin contact immediately after birth or high caseload of birth. Further, qualitative assessment is needed to identify the factors for non-adherence to standards. To improve skin to skin contact immediately after birth there is a need to build capacity on clinical standards. We have mentioned this in page 19, lines 8-9.

**Comment 2#.** A possible impact of observation on care givers behaviour could be mentioned as a study limitation and possible bias

**Response.** We have provided the possible bias of observers reporting in limitation section.

**Response to second reviewer’s comment**

**Comment 3#.** A general problem remains: This study seems more to describe a health care system in which evidence based neonatal basic care is almost absent, than a voluntarily applied mistreatment of newborn infants. The title might be reconsidered.

**Response.** We agree that the study describes the non-adherence to standard care for newborn at the time of birth within a health system context. We define mistreatment as the care which deviates from the standard of care.

**Comment 4#.** This study is important as it shows an absence of knowledge regarding neonatal care and an important need for improvement. However, to declare it as mistreatment as suggested in the second paragraph of the Background chapter, might be questionable. The main information of this study is comprised in Table 1 and the description in the first paragraph of the discussion (page 18, line 3 - 9).

**Response.** This study provides new evidence on the deviation of clinical practice for newborn during childbirth. We have defined mistreatment in the method section, page 10, lines 9-10.

**Comment 5#.** Interpretation of the results are not always clear or are lacking. Therefore several questions remain: # Sex of infants: In lines 5ff of page 17 is stated : « There was no association between mistreatment with woman’s literacy, parity, and newborn sex ». In line 13 is stated: « Female newborns were more likely to be mistreated than male newborns. » What is now true?

**Response.** There was a typo. In page 17, line 6 it should be stated “There was no association between mistreatment with woman’s literacy, parity and premature birth” rather than ”…. newborn sex”. We have now revised it.

**Comment 6#.** Interpretation of the results are not always clear or are lacking. After adjustment, there is a change of the risk being mistreated for infants of mothers > 35 years, initially higher, after adjustment lower risk. Explanation is still missing for this risk change.

**Response.** we have now added the explanation on the change in the results on association of between mistreatment with socio-demographic and neonatal characteristics in limitation section in page 21, lines 20-23 as “Fifth, due to the high level of heterogeneity in process of care for newborns by hospitals, the association between mistreatment with socio-demographic and neonatal characteristics (age, literacy, parity and prematurity) changed after adjusting hospital level heterogeneity.”

**Comment 7#.** Interpretation of the results are not always clear or are lacking. Page 15 lines 14/15 : Statement about mistreatment of women, however, the study is about their newborns. This should be clarified.

**Response.** Thank you for the observation. We have now improved it as “Infants born to …..”

**Comment 8#.** Interpretation of the results are not always clear or are lacking. There is a statement that newborns of mothers of less advantaged ethnic groups are at higher risk for mistreatment. However, there is no explanation why newborns of mothers of the ‘janjati’ ethnic group, although less advantaged are at lower risk for mistreatment. Hypothèses?

**Response.** We have now explained it in page 20, lines 13-20. “The study found that of the ethnic group, only newborn born to women janjati ethnic group are at a relative risk of mistreatment in reference to relatively advantaged ethnic group. The study did not find any association with other relatively disadvantaged group (dalit, madeshi and other disadvantaged). This might be due to health worker’s attitude towards women from janati ethnic group or might be health workers were from other ethnic group and could not provide care culturally appropriate care. Further exploration will be required to understand the provider and client dynamics based on ethnicity.”

**Comment 9#.** What exactly is the content of the parental information? This is important, as one of the major finding of this study is the non-consented treatment of the newborn infants. Although it is stated in the revised manuscript that consent was given to observation and not for treatment, this difference might be difficult to be understood by mothers and also by health care workers. One conclusion of this point is to be made: information of mothers about neonatal care should be improved, but for this being possible, health care workers should be formed about basic skills and trained in their application.

**Response.** We notice the confusion on the consent of parental information. There are two kind of consent for parental information. For, the research ethics purpose, consent was taken by our independent observers from women to observe them during clinical care process. For the clinical care purpose, observation was done whether health workers informed women and consented for any additional care provided to newborns. We agree to improve parental information, health workers skills needs to be developed. We have added a sentence in page 20, lines 4-6 as “Health workers will require training to improve their skills to inform women and consent for care being provided to newborn infants.”

**Comment 10#.** Caesarean section and clear definition of the denominator: it is still not clearly written that there are only newborns after vaginal birth included. Page 14 line 9ff : It is stated: “Of the total 39,652 women admitted for delivery in the hospitals during the study period, 34,125 of them consented and were enrolled in the study. Among their newborns, 31,804 breathed spontaneously at birth and were eligible for inclusion in the study (figure 1).” However: there was a caesarean section rate of 18 – 27%. Numbers have to be explained.

**Response.** Thank you. As explained in the inclusion criteria in method section, page 9 lines 1-2. The study participants were “all vaginally born newborns who were spontaneously breathing at birth…” In the setting in page 8, lines 18-19 we provide the context that in these four hospitals the caesarean section rate was 18-27%. We have clarified in the result section page 14, line 9-11 as “ Of the total 39,652 women admitted in labour room for vaginal delivery …../ Among their vaginally born newborns….”

**Comment 11#.** The statement on page 19, lines 13ff has to be changed : « The use of unnecessary interventions such as suctioning to a spontaneously breathing newborns has been observed in previous study on neonatal resuscitation[26]. Reducing this intervention would also allow delayed cord clamping, early initiation of breast-feeding, and immediate skin to skin contact. » The suctioning is not responsible for dealyed breast-feeding and immediate skin to skin contact.

**Response.** We have now removed the sentence.

**Comment 12#.** Taken together: The results of the highly performed statistics are not sufficiently explained. The important findings of this study are the fact that basic neonatal care is mainly absent in the four major maternity wards of Nepal and that there is a tremendous need for training of neonatal care algorithms and skills.

**Response.** We used advanced statistics to explain the phenomenon of mistreatment to newborns. We have explained the statistics performed in page 13, line 10-22. We agree than there is a need to improve the clinical care process in four hospitals of Nepal and have now added it in page 20, lines 7-9 as “Health workers will require training to improve their skills to inform women and consent for care being provided to newborn infants.”
